# Inhibition of Tet1- and Tet2-mediated DNA demethylation promotes immunomodulation of periodontal ligament stem cells

**DOI:** 10.1038/s41419-019-2025-z

**Published:** 2019-10-14

**Authors:** Tingting Yu, Dawei Liu, Ting Zhang, Yanheng Zhou, Songtao Shi, Ruili Yang

**Affiliations:** 1 Department of Orthodontics, Peking University School and Hospital of Stomatology, #22 Zhongguancun South Avenue, 100081 Beijing, China; 2 Department of Anatomy and Cell Biology, School of Dental Medicine, University of Pennsylvania, Philadelphia, PA 19104 USA; 3 South China Center of Craniofacial Stem Cell Research, School of Guanghua Dental Medicine, Sun Yat-sen University, #74 Zhongshan 2 Road, Guangzhou, Guangdong 510080 China

**Keywords:** Apoptosis, Mesenchymal stem cells

## Abstract

Periodontal ligament stem cells (PDLSCs) possess great potential for clinical treatment of immune diseases due to their extensive immunomodulatory properties. However, the underlying mechanisms that govern the immunomodulatory properties of mesenchymal stem cells (MSCs) are still not fully elucidated. Here, we show that member of the Ten-eleven translocation (Tet) family, a group of DNA demethylases, are capable of regulating PDLSC immunomodulatory functions. Tet1 and Tet2 deficiency enhance PDLSC-induced T cell apoptosis and ameliorate the disease phenotype in colitis mice. Mechanistically, we found that downregulation of Tet1 and Tet2 leads to hypermethylation of *DKK-1* promoter, leading to the activation of WNT signaling pathway and therefore promoting Fas ligand (FasL) expression, which results in elevated immunomodulatory capacity of PDLSCs. These results reveal a previously unrecognized role of Tet1 and Tet2 in regulating immunomodulation of PDLSCs. This Tet/DKK-1/FasL cascade may serve as a promising target for enhancing PDLSC-based immune therapy.

## Introduction

The Ten-eleven translocation (Tet) family is a group of recently identified demethylases capable of modifying DNA by hydroxylating 5-methylcytosine (5-mC) to 5-hydroxymethylcytosine (5-hmC)^1–3^. This discovery revealed a novel mechanism by which Tet enzymes regulate DNA demethylation. Three Tet family members (Tet1, Tet2, and Tet3) show distinct expression patterns depending on cell or tissue type and developmental stage^4,5^. For instance, Tet1 is highly expressed in mouse embryonic stem cells (ESCs); upon ESC differentiation, its expression level is significantly decreased. However, some terminally differentiated cells, such as Purkinje neurons, also show upregulated Tet1 expression^2,6,7^. Emerging evidences indicate that Tet-mediated DNA demethylation contributes to normal growth and developmental processes as well as disease development. Alteration of Tet expression has been linked to tumorigenesis, neural disorders, and T cell-related immune disorders^8^. Our previous studies showed that Tet1 and Tet2 play a crucial role in maintaining bone marrow mesenchymal stem cell (BMMSC) functions and bone homeostasis. Tet1 and Tet2 depletion resulted in osteopenia and impairment of BMMSC differentiation^9^, suggesting that the Tet family is able to modulate MSC biological function. Moreover, we have found that Tet1 and Tet2 help to maintain immune homeostasis via regulating regulatory T (Treg) cell function^10^. Whether Tet1/Tet2-mediated DNA demethylation modulates MSC-mediated immunomodulation remains unclear.

Periodontal ligament stem cells (PDLSCs), isolated from periodontal ligament tissue, possess unique immunomodulatory capacity^11^. PDLSCs interact with different immune cells, for example, suppressing T cell proliferation^12^ and upregulating Tregs^13^. After transplantation in a periodontitis minipig model, PDLSCs were able to modulate the local immune microenvironment and result in periodontal tissue regeneration^14^. Therefore, it is possible to use PDLSCs to treat inflammatory diseases^14,15^, such as periodontal disease.

In the present study, we demonstrated that Tet1/Tet2-mediated DNA demethylation is capable of regulating the immunomodulatory function of PDLSCs. Tet1 and Tet2 deficiency enhances PDLSC-induced T cell apoptosis and ameliorates the disease phenotype in colitis mice.

## Results

### Human PDLSCs express Tet1 and Tet2

**Fig. 1:**
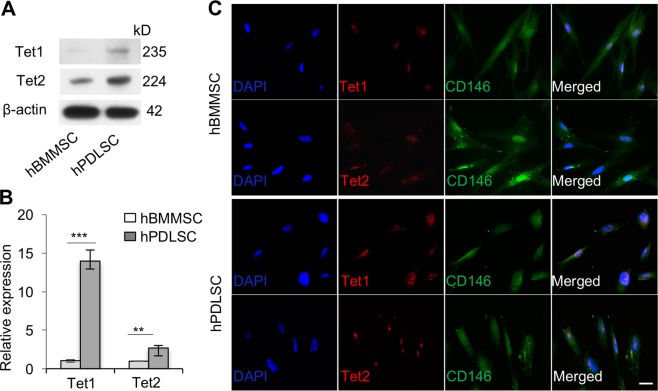
Human periodontal ligament stem cells (hPDLSCs) express Tet1 and Tet2. **a**, **b** Both human bone marrow mesenchymal stem cells (hBMMSCs) and hPDLSCs expressed Tet1 and Tet2, as assessed by Western blotting (**a**) and qPCR (**b**). **c** Immunocytofluorescent staining showed that CD146-positive hPDLSCs express Tet1 and Tet2. Scale bar, 50 µm. ***P < 0.001, **p < 0.01; *p* values were calculated using two-tailed Student’s *t* test (mean ± SD)

We found that human PDLSCs (hPDLSCs) isolated from human periodontal ligament expressed elevated levels of Tet1 and Tet2, as assessed by Western blotting and real-time polymerase chain reaction (qPCR), when compared to human BMMSCs (hBMMSCs) (Fig. 1a, b). Immunostaining showed that MSC marker CD146 was co-expressed with Tet1 and Tet2 in hPDLSCs (Fig. 1c). These data indicate that PDLSCs express certain levels of Tet1 and Tet2.

### Tet1 and Tet2 regulate PDLSC-mediated immunomodulation

**Fig. 2:**
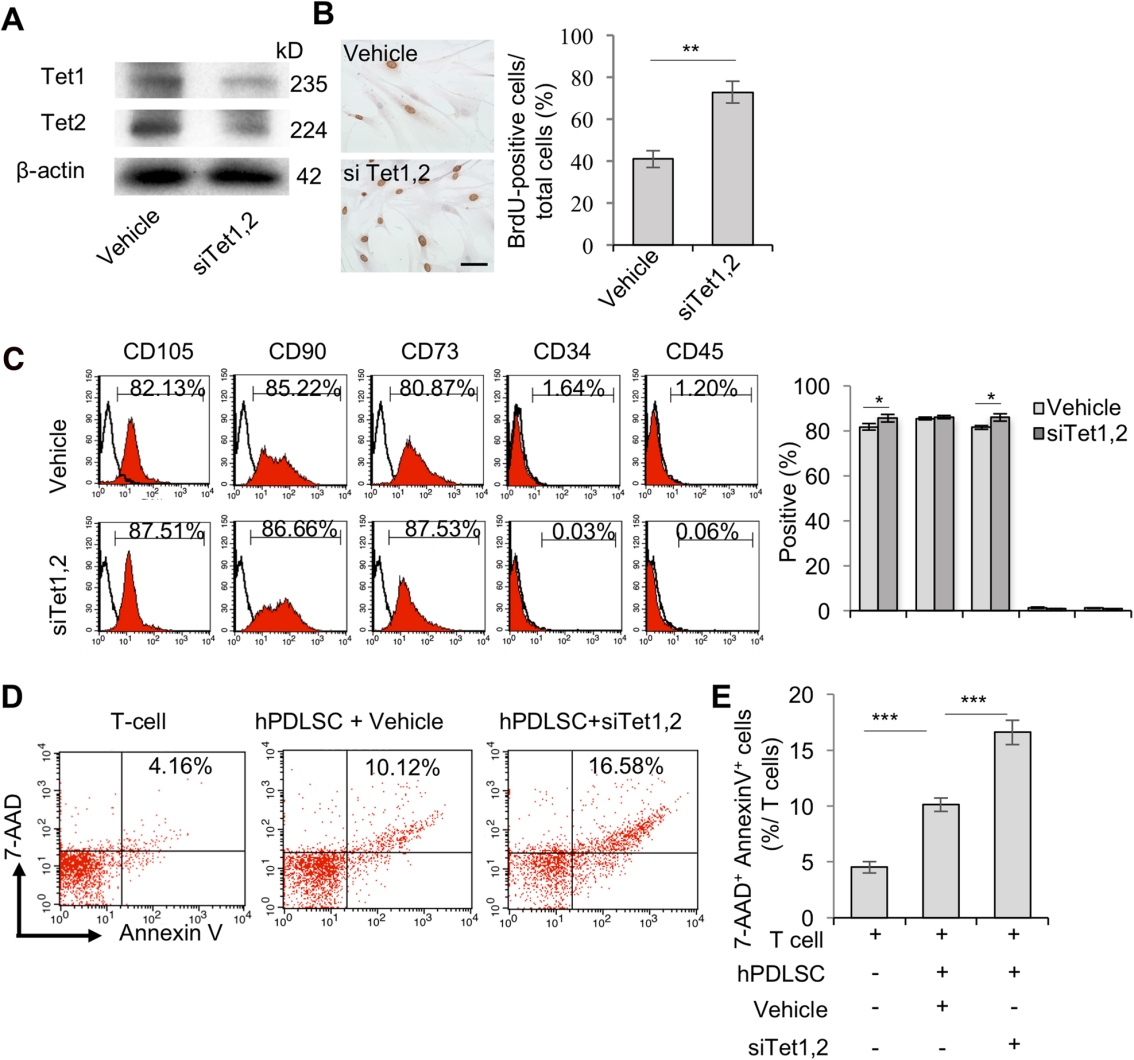
Tet regulates hPDLSC-mediated immunomodulation. **a** Western blot analyzed the efficiency of Tet1 and Tet2 small interfering RNA (siRNA) knockdown in hPDLSCs. **b** BrdU labeling assay was performed to show elevated proliferation rates of Tet1 and Tet2 siRNA-treated hPDLSCs. Scale bar, 50 µm. **c** Flow cytometry was used to analyze the expression of CD105, CD90, CD73, CD34, and CD45 in control and Tet1 and Tet2 siRNA-treated hPDLSCs. **d**, **e** In vitro coculture showed a significantly increased capacity of Tet1 and Tet2 siRNA-treated hPDLSCs to induce T cell apoptosis (AnnexinV^+^7AAD^+^) of T cells. ***P < 0.001, **p < 0.01, and *p < 0.05; *p* values were calculated using two-tailed Student’s *t* test (mean ± SD)

To investigate whether Tet1 and Tet2 mediate DNA demethylation that may regulate the multi-lineage differentiation and immunomodulation capacities of PDLSCs, we knocked down Tet1 and Tet2 expression in PDLSCs by using small interfering RNAs (siRNAs) (Fig. 2a). Bromodeoxyuridine (BrdU)-labeling assays showed that knocking down Tet1 and Tet2 in PDLSCs led to upregulated proliferation when compared with the control group (Fig. 2b). Flow cytometric analysis showed that MSC surface markers, including CD105 and CD73, were significantly elevated in Tet1/Tet2 siRNA-treated PDLSCs compared with control PDLSCs, but not CD90. The hematopoietic lineage markers, CD34 and CD45, were absent in Tet1/2 siRNA-treated PDLSCs, similar to the observations of control PDLSCs (Fig. 2c). Furthermore, we cultured PDLSCs and Tet1/Tet2 siRNA-treated PDLSCs under osteogenic and adipogenic differentiation condition and found that Tet1 and Tet2 deficiency led to significantly decreased osteogenic (Fig. S1a, b) and adipogenic (Fig. S1c, d) differentiation potential when compared to the control PDLSCs.

Next, to evaluate the immunomodulatory properties of PDLSCs, we co-cultured PDLSCs with T cells and found that PDLSCs were capable of inducing T cell apoptosis^16^. Moreover, Tet1/Tet2 siRNA-treated PDLSCs had a significantly elevated capacity to induce AnnexinV^+^7AAD^+^ double-positive T cell apoptosis, when compared to the control PDLSCs (Fig. 2d, e). These results indicate that the inhibition of Tet1 and Tet2 promotes PDLSCs’ immunomodulatory capacity by inducing T cell apoptosis.

### Inhibition of Tet1 and Tet2 enhances therapeutic effect of PDLSCs in treating colitis

**Fig. 3:**
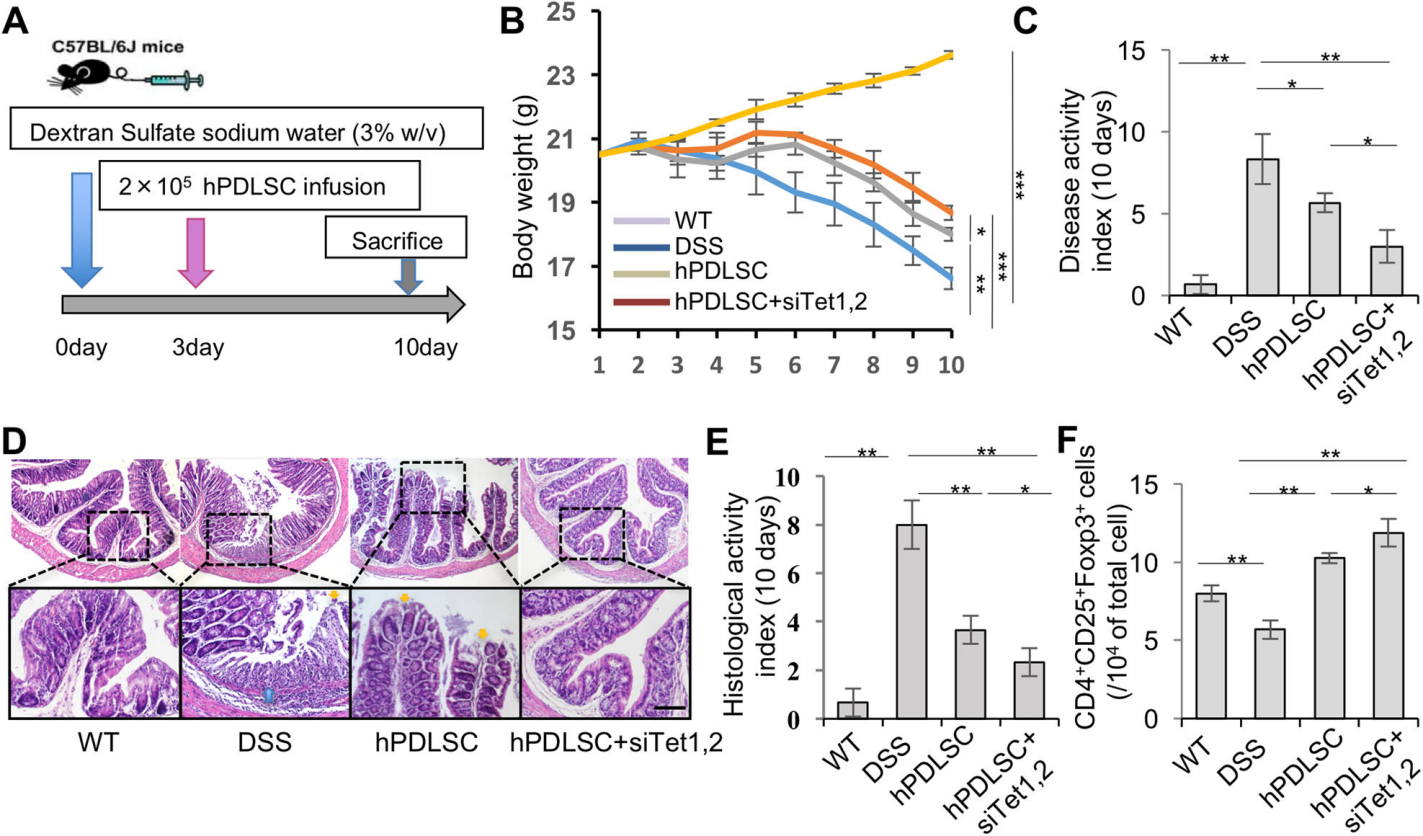
Inhibition of Tet1 and Tet2 enhances PDLSC-mediated amelioration of disease phenotype in colitis mice. **a** Schema showing PDLSC transplantation for treating colitis mice. **b–e** Knockdown of Tet1 and Tet2 by siRNA treatment elevated the immunomodulatory capacity of PDLSCs, as assessed by amelioration of the reduced body weight (**b**), a decreased disease activity index (DAI) (**c**), and alleviation of the colitis histologic activity index (HAI). Scale bar in **d**, 100 µm. **f** Flow cytometry analysis showed that the Treg level significantly decreased in colitis mice compared to control littermates. After PDLSC treatment, the Treg level was significantly elevated, and the Tet1 and Tet2 siRNA-treated PDLSC group showed a higher Treg level than the group with control PDLSCs. ***P < 0.001, **p < 0.01, *p < 0.05; *p* values were calculated using two-tailed Student’s *t* test (mean ± SD)

The ability of MSCs to modulate immune response is one of their most important characteristics^17,18^. To further assess the role of Tet1 and Tet2 in immunomodulation by PDLSCs, we compared the immunotherapeutic effects of control and Tet1/Tet2 siRNA-treated PDLSCs in experimental colitis mice. C57BL/6J mice were orally administered 3% dextran sodium sulfate (DSS) for 10 days to establish acute colitis. On day 3, colitis mice were treated with Tet1/Tet2 siRNA-treated PDLSCs or control PDLSCs by systemic transplantation through tail vein, followed by sacrifice of the mice on day 10 to collect samples for evaluation (Fig. 3a).

Consistent with previous reports^19^, DSS-induced colitis mice lost weight at a sustained rate and exhibited bloody diarrhea/loose feces, which were characterized by an overall evaluation of their condition using the established disease activity index (DAI). Infusion of either Tet1/Tet2 siRNA-treated PDLSCs or control PDLSCs partially restored the reduced body weight of the colitis mice and decreased their DAI scores. Furthermore, Tet1/Tet2 siRNA pretreatment was able to enhance the ability of PDLSCs to restore the reduced body weight (Fig. 3b) and decreased the DAI scores (Fig. 3c). Histologically, localized inflammatory cell infiltration, epithelial ulceration, and impairment of crypt architecture were observed in the intestines of DSS-induced colitis mice. Systemic infusion of either Tet1/Tet2 siRNA-treated PDLSCs or control PDLSCs could recover impaired intestinal structure. However, Tet1/Tet2 siRNA-treated PDLSCs demonstrated a superior ability to eliminate the inflammatory cells and recover the epithelial structure, as assessed by the histologic activity index (HAI) (Fig. 3d, e). Moreover, decreased number of CD4^+^CD25^+^Foxp3^+^ Tregs were observed in the mice with colitis at day 10 post-DSS induction (Fig. 3f). Infusion of control PDLSCs elevated the number of Treg cells, and Tet1/Tet2 siRNA pretreatment enhanced this effect. Taken together, these experimental data indicate that Tet1 and Tet2 inhibition results in an elevated capacity for PDLSC-mediated immunomodulation.

### Tet1 and Tet2 regulate FasL expression through Wnt/β-catenin pathway in PDLSCs

**Fig. 4:**
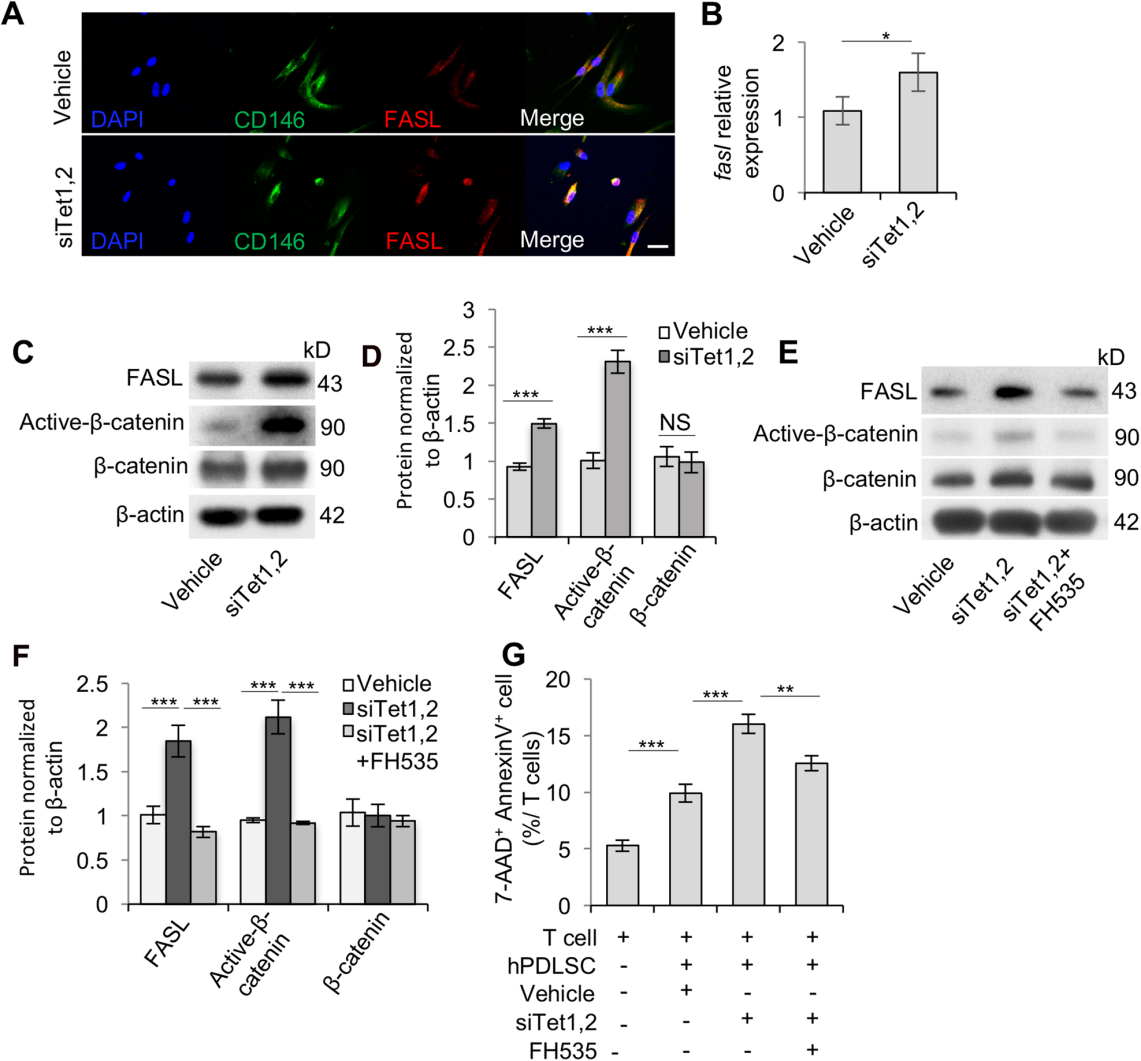
Tet1 and Tet2 regulate FasL expression in hPDLSCs. **a** Immunocytofluorescent staining showed that CD146-positive hPDLSCs express FASL. Scale bar, 50 µm. **b** qPCR analysis showed the significantly increased expression level of *Fasl* in Tet1 and Tet2 siRNA-treated hPDLSCs compared to control hPDLSCs. **c**, **d** Western blot analysis showed increased levels of FASL and active β-catenin in Tet1 and Tet2 siRNA-treated hPDLSCs compared to the control hPDLSCs. **e**, **f** β-Catenin inhibitor (FH535) treatment decreased the levels of FASL and active β-catenin in Tet1 and Tet2 siRNA-treated hPDLSCs. **g** In vitro coculture showed β-catenin inhibitor (FH535) treatment decreased the capacity of Tet1 and Tet2 siRNA-treated hPDLSCs to induce apoptosis (AnnexinV^+^7AAD^+^) of T cells. ***P < 0.001, **p < 0.01, *p < 0.05; *p* values were calculated using two-tailed Student’s *t* test (mean ± SD)

To explore the underlying mechanism of how Tet1 and Tet2 regulate immunomodulation of PDLSCs, we examined the levels of FasL, a key member of the apoptosis pathway, in PDLSCs. It has been reported that FasL is related to MSCs’ immunomodulatory ability, as a previous study demonstrated that FasL in MSCs induced T cell apoptosis and immune tolerance^20^. Thus, in order to confirm that PDLSCs express FasL, we used double immunostaining and found that CD146-positive PDLSCs expressed FasL (Fig. 4a). To investigate whether Tet1 and Tet2 modulate the FasL expression level, Western blot and qPCR analysis were used, and it revealed that FasL was significantly elevated in Tet1/Tet2 siRNA-treated PDLSCs (Fig. 4b, c), indicating that the inhibition of Tet1 and Tet2 upregulates FasL expression and results in an elevation of MSC-based immunomodulatory ability. Next, to evaluate whether FasL is a direct target of Tet1 and Tet2, we used the Methprimer software to detect the promoter of FasL and found that the *FasL* promoter lacks CpG island, which indicates that Tet1 and Tet2 may not prefer to bind to the promoter of FasL^21,22^. Thus, we continued searching for potential molecules that may connect Tet with FasL. Since β-catenin could serve as a transcriptional factor to regulate FasL expression^23,24^, we next examined the Wnt/β-catenin expression levels in Tet1/Tet2 siRNA-treated PDLSCs. Western blotting showed that the expression level of active β-catenin (non-phosphorylated) was indeed significantly elevated, although the total level of β-catenin was not significantly elevated in Tet1/Tet2 siRNA-treated PDLSCs (Fig. 4c, d). The expression levels of active β-catenin and FasL were significantly decreased after the treatment of β-catenin inhibitor (FH535) (Fig. 4e, f). When co-cultured with T cells, Tet1/Tet2 siRNA-treated PDLSCs gained an elevated capacity to induce AnnexinV^+^7AAD^+^ double-positive T cell apoptosis when compared to the untreated group, but the elevated induction of apoptosis could be abrogated by β-catenin inhibitor (FH535) treatment (Fig. 4g). These results suggest that Tet1 and Tet2 act as upstream regulators of Wnt/β-catenin signaling to modulate FasL expression, which in turn regulates the PDLSC-mediated immunomodulatory capacity.

Furthermore, besides FasL, many other target genes can be regulated by β-catenin. Therefore, we analyzed Notch and tumor growth factor-β signaling-related genes, which have been identified as the downstream of WNT/β-catenin pathway^25,26^. Western blot analysis showed that the expression level of P-smad3 was decreased, while Notch1 and Notch2 showed an elevated expression in Tet1/Tet2 siRNA-treated PDLSCs compared with the control group (Fig. S2). These results indicated that Tet might be able to regulate other WNT-associated genes.

### Tet1 and Tet2 serve as demethylases on *DKK-1* promoter to regulate FasL expression in PDLSCs

**Fig. 5:**
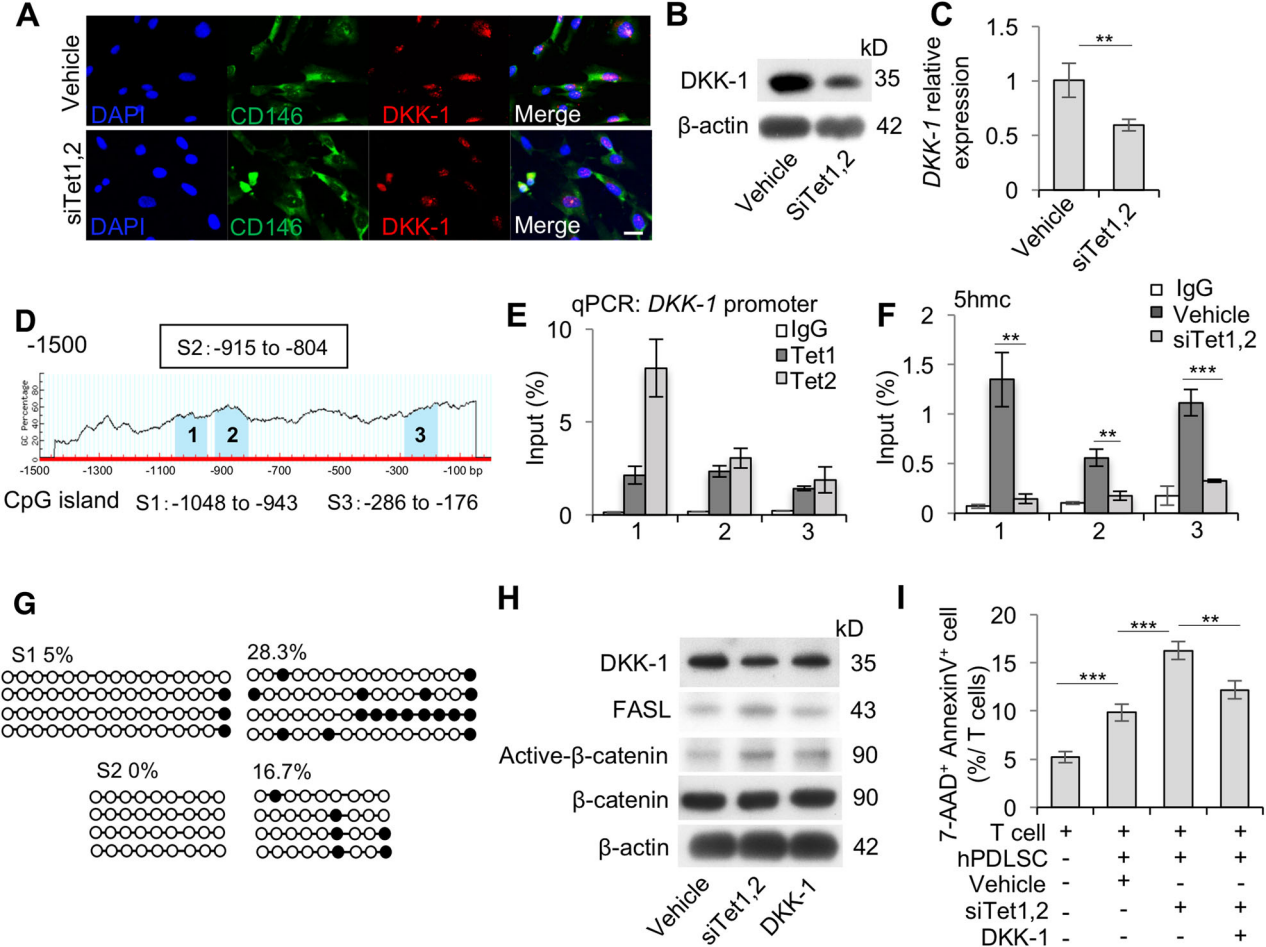
Tet1 and Tet2 regulate FASL through demethylation of DKK-1. **a** Immunocytofluorescent staining showed that CD146-positive hPDLSCs express DKK-1. Scale bar, 50 µm. **b** Western blot analysis showed decreased levels of DKK-1 in Tet1 and Tet2 siRNA-treated hPDLSCs compared to the control hPDLSCs. **c** qPCR analysis showed significantly decreased expression of *DKK-1* in Tet1 and Tet2 siRNA-treated hPDLSCs compared to the control hPDLSCs. **d** The CpG island of *DKK-1*. **e** Tet1 and Tet2 binding on the promoter of *DKK-1* in hPDLSCs, as assessed by ChIP-qPCR. IgG was used as a control. **f** Enrichment of 5-hmC in the *DKK-1* promoter decreased in Tet1 and Tet2 siRNA-treated hPDLSCs. **g** OxBS-sequencing analysis showed that Tet1 and Tet2 siRNA-treated hPDLSCs displayed elevated methylation in the promoter of DKK-1 locus compared to control hPDLSCs. Empty dots indicate unmethylated GpGs; black dots indicate methylated CpGs. **h** DKK-1 treatment increased the level of DKK-1 and decreased the expression levels of FASL and active β-catenin in Tet1 and Tet2 siRNA-treated hPDLSCs, assessed by Western blot. **i** In vitro coculture showed that DKK-1 treatment decreases the capacity of Tet1 and Tet2 siRNA-treated hPDLSCs to induce apoptosis (AnnexinV^+^7AAD^+^) of T cells. ***P < 0.001, **p < 0.01; *p* values calculated using two-tailed Student’s *t* test (mean ± SD)

**Fig. 6:**
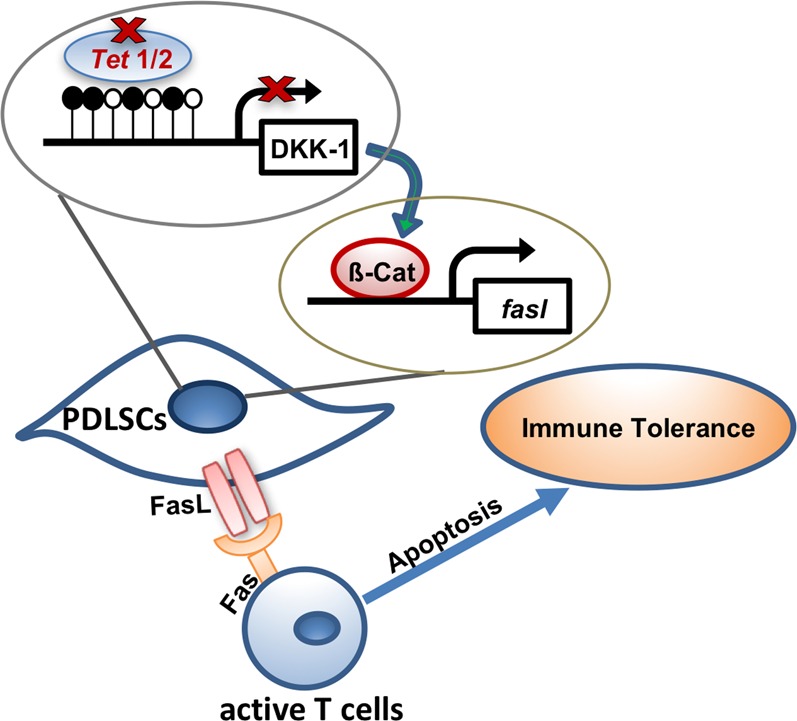
Schema diagram of the immunomodulatory property of PDLSCs regulated by Tet1 and Tet2-mediated DNA demethylation

To investigate how Tet1/Tet2 regulate Wnt/β-catenin signaling, we examined the level of DKK-1, an upstream inhibitor of the WNT pathway^27^, and found that DKK-1 was downregulated in Tet1/Tet2 siRNA-treated PDLSCs. Immunofluorescence staining showed co-localization of DKK-1 with MSC surface marker CD146 (Fig. 5a). Western blotting (Fig. 5b) and qPCR (Fig. 5c) showed that the expression level of DKK-1 was significantly decreased in Tet1/Tet2 siRNA-treated PDLSCs compared to the control group. As previous studies indicated that Tet1 and 5-hmC prefer to co-localize at CpG-rich locus in the promoter transcriptional start sites^21,22^, we examined the promoter region of *DKK-1* (DKK-1 −1500 to 0, *Homo sapiens* chromosome 10, GRCh38 52,312,781 to 52,314,281) by using the Methprimer software; three CpG islands (DKK-1 −1048 to −943, −915 to −804, −286 to −176) were detected (Fig. 5d). To demonstrate if Tet1 and Tet2 are able to regulate *DKK-1* directly, chromatin immunoprecipitation-qPCR (ChIP-qPCR) analysis were used to verify that Tet1 and Tet2 were able to bind to the CpG islands of the *DKK-1* promoter (Fig. 5e). We next examined whether inhibition of Tet1 and Tet2 affected the enrichment of 5-hmC level at the *DKK-1* promoter. Hydroxymethylated DNA immunoprecipitation-qPCR (hMeDIP-qPCR) analysis showed that Tet1/Tet2 siRNA-treated PDLSCs displayed a significantly decreased 5-hmC level compared to control PDLSCs on the three CpG island sites of the *DKK-1* promoter (Fig. 5f). Oxidative bisulfite-sequencing (OxBS) sequencing analysis also showed an elevated methylation status in the promoter of *DKK-1* locus in Tet1/Tet2 siRNA-treated PDLSCs compared to control PDLSCs (Fig. 5g). These results demonstrated that Tet1 and Tet2 are able to directly regulate *DKK-1* expression through DNA demethylation. Next, we added DKK-1 to Tet1/Tet2 siRNA-treated PDLSCs and found that exogenous DKK-1 could block the increased expression of active β-catenin and FasL that was induced by Tet1/Tet2 siRNA treatment (Fig. 5h). When co-cultured with T cells, Tet1/Tet2 siRNA-treated PDLSCs gained an elevated capacity to induce AnnexinV^+^7AAD^+^ double-positive T cell apoptosis when compared to the untreated group, but that effect could be abrogated by DKK-1 treatment (Fig. 5i). Taken together, these findings indicate that the inhibition of Tet1 and Tet2 could promote PDLSC-mediated immunomodulation ability through regulating *DKK-1* demethylation (Fig. 6).

## Discussion

Tet-mediated conversion of 5-mC to 5-hmC has been proposed as the initial step in active DNA demethylation, which plays an important role in maintaining tissue homeostasis^10,28,29^. Inhibition of Tet leads to deficiency of Tregs^28^ and myeloid invariant natural killer T cells, as well as B cell malignancy^30–32^. Our previous study demonstrated that Tet1 and Tet2 are required to maintain bone homeostasis via regulating the osteogenic differentiation of BMMSCs^9,33^. Here, we showed that inhibition of Tet1 and Tet2 led to decreased osteogenic and adipogenic differentiation of PDLSCs (Fig. S1). Moreover, we showed that Tet1 and Tet2 deficiency promoted the proliferation of hPDLSCs (Fig. 2b), in agreement with our previous finding in mouse BMMSCs^9^. However, based on studies on umbilical cord (UC)-derived MSCs, loss of Tet1/2 can inhibit the ability of MSCs to replicate^34^, which implies that different effects of Tet1/2 on MSC proliferation may be regulated by specific molecules^9,34,35^.

MSCs possess great potential for treating immune diseases due to their extensive immunomodulatory properties^16,20^. Whether Tet participates in regulating MSC immunomodulation has remained unknown. In the present study, we demonstrated that inhibition of Tet1 and Tet2 promoted PDLSC-induced T cell apoptosis (Fig. 2d, e). Importantly, when PDLSCs were systematically infused into DSS-induced colitis mice, Tet1/Tet2-downregulated PDLSCs showed significantly increased therapeutic effects (Fig. 3). These results may provide a promising method to promote MSC-mediated therapeutic efficacy.

The multi-potent and immunomodulatory properties of MSCs make them a valuable cell source for regeneration and immune therapy^36,37^. The key issue in MSC biology is to illustrate the underlying mechanisms that control MSCs’ multiple distinct functions. In the present study, we demonstrated that Tet deficiency led to inhibition of PDLSC differentiation toward osteogenic and adipogenic lineages (Fig. S1), similar to the observations made concerning BMMSCs in our previous report^9^. Interestingly, we also found that inhibition of Tet1 and Tet2 could elevate PDLSC immunomodulatory potential (Figs. 2 and 3), which indicates that *Tet1* and *Tet2* might serve as switch genes to control MSC differentiation and immunomodulation. Downregulation of Tet1 and Tet2 resulted in differentiation deficiency as well as upregulation of immunomodulation ability in PDLSCs. However, based on our current understanding, more investigation is required to further illustrate the mechanisms of how Tet regulates MSCs’ distinct functions in differentiation and immunomodulation.

Mechanistically, we identified a novel role of Tet1 and Tet2 in binding directly to the *DKK-1* promoters, and further identified that they act as an upstream regulator of Wnt/β-catenin pathway to modulate FasL expression (Fig. 6). Previous studies demonstrated that Wnt pathway inhibitor DKKs could be inactivated by DNA methylation in cancer cells to sustain tumor development^38,39^, which can be reversed by demethylation activity on the *DKK* promoter region^40^. In the present work, we reported that Tet1 and Tet2 bind to the promoter of *DKK-1* to maintain its hypomethylation in PDLSCs. It is known that MSCs are able to induce T cell apoptosis via Fas/FasL pathway, resulting in upregulation of Tregs and immune tolerance^20^. β-Catenin could directly bind to the *FasL* promoter to drive gene expression at the transcriptional level^23^. Based on our findings, downregulation of Tet1 and Tet2 led to repression of *DKK-1* by DNA methylation modification, which results in activation of Wnt pathway and further promotes FasL expression in PDLSCs to enhance their immunomodulatory ability. This study provided evidence to link Tet1/Tet2, which are DNA demethylases, to MSC-mediated immunomodulation. It has been reported that Tet is able to modulate *Foxp3* demethylation in T cells to affect immune homeostasis^10^ and could directly target the promoters of microRNAs in cancer cells^41^. Except Fas/FasL pathway, we showed that the expression of P-smad3, Notch1, and Notch2 were altered after siRNA knockdown; however, whether PDLSC-mediated immunomodulation could be modulated by other molecules needs further investigation to illustrate the detailed mechanism.

In summary, we demonstrated that Tet1 and Tet2 participate in the regulation of MSC-mediated immunomodulation. Downregulation of Tet1 and Tet2 in PDLSCs improves their immunomodulatory functions by inhibiting *DKK-1* expression through hypermethylation. Tet1 and Tet2 knockdown pretreatment in PDLSCs may be a promising approach to enhance MSC-mediated immune therapeutic effects.

## Materials and Methods

### Antibodies

Tet1 (ab191698), Tet2 (ab94580), and CD146 (ab24577) antibodies were purchased from Abcam (Cambridge, MA, USA). Phospho-β-catenin (Ser552) and β-catenin (15B8) antibodies were purchased from Invitrogen (Carlsbad, CA, USA). FASL (SC-33716) and DKK-1 (SC-374574) antibodies were purchased from Santa Cruz Biotechnology (Dallas, TX, USA). β-Actin antibody (A1978) was purchased from Sigma-Aldrich (St. Louis, MO, USA). Anti-CD4-PerCP (550765) and anti-CD25-APC (561048) were purchased from BD Biosciences (San Jose, CA, USA). Anti-CD3 (100208) and anti-CD28 (102112) were purchased from BioLegend (SanDiego, CA, USA)

### Isolation and culture of hPDLSCs

Periodontal ligament tissues were obtained from healthy patients (18–25 years old) without any history of periodontal disease who were underdoing orthodontic extraction. The protocol of PDLSC isolation and cultivation was followed according to a previous publication by Seo et al.^42^; the procedure was approved by the Ethical Guidelines of Peking University (PKUSSIRB-201311103). Passage 3 PDLSCs were used in all experiments.

### siRNA and chemical treatment

For siRNA transfection, PDLSCs were cultured under reduced serum medium (Opti-MEM, Gibco), and Tet1 siRNA (sc-90457), Tet2 siRNA (sc-88934), or control vehicle siRNA (sc-37007) (Santa Cruz Biotechnology) were treated with Lipofectamine reagent (Invitrogen), according to the manufacturer’s instructions. For chemical reagent treatments, serum-starved PDLSCs were treated with 10 μM Wnt/β-catenin inhibitor (FH535; Cayman Chemical), or human DKK-1 (PeproTech). Treated cells were collected for further experiments.

### Western blotting

Mammalian protein extraction reagent (Thermo, Rockford, IL, USA) was used to lyse total protein. The protein was applied and separated on 4 to 12% NuPAGE gel (Invitrogen), which were then transferred to nitrocellulose membranes (Millipore, Billerica, MA, USA). Membranes were blocked with 5% nonfat dry milk and 0.1% Tween-20 for 1 h, which were then incubated with the primary antibodies at 4 °C overnight. We used horseradish peroxidase-conjugated secondary antibody (Santa Cruz Biotechnology; 1:10,000) to treat the membranes for 1 h. By using Super Signal West Pico Chemiluminescent Substrate (Thermo) and BioMax film (Kodak, USA), the immuno-reactive proteins were detected.

### Real-time PCR

Total RNA was isolated using miRNeasy Mini Kit (Qiagen, Valencia, CA, USA) according to the manufacturer’s instructions. SuperScript III Reverse Transcriptase (RT) Kit (Invitrogen) was used to prepare the complementary DNA. qPCR was performed using SYBR Green Supermix (Bio-Rad, Hercules, CA, USA) and gene-specific primers. Detection was performed on a CFX96 Real-Time PCR System (Bio-Rad).

### Immunofluorescent staining

PDLSCs (1 × 10^3^/well) were cultured in chamber slides (Nunc, Rochester, NY, USA) and then fixed with 4% paraformaldehyde (PFA). The cells were incubated with primary antibodies at 4 °C overnight, and then treated with Alexa Fluor 568 or Alexa Fluor 488-conjugated secondary antibody (1:200, Invitrogen) for 1 h. Finally, slides were mounted with Vectashield mounting medium containing 4′,6-diamidino-2-phenylindole (DAPI) (Vector Laboratories, Burlingame, CA, USA).

### T lymphocytes apoptosis assay

A total of 0.2 × 10^6^ PDLSCs (with or without Tet1/2 siRNA treatment) were seeded on a 24-well culture plate (Corning) containing Dulbecco’s modified Eagle’s medium (Lonza, Basel, Switzerland) with 10% heat-inactivated fetal bovine serum, 10 mM HEPES, 50 μM 2-mercaptoethanol, 1 mM sodium pyruvate (Sigma-Aldrich), 1% non-essential amino acid (Cambrex, East Rutherford, NY, USA), 2 mM l-glutamine, 100 U/ml penicillin, and 100 μg/ml streptomycin. Activated T cells (1 × 10^6^/well), which were isolated from mouse spleen, were pre-stimulated with plate-bound anti-CD3ε and anti-CD28 (2 μg/ml each; BD Bioscience) for 2 days, and then loaded directly on PDLSCs. After 24 h of coculture, CD3 antibody and AnnexinV Apoptosis Detection Kit I (BD Bioscience) were used to detect apoptotic T cells using a FACS flow cytometer.

### Cell proliferation assay

PDLSCs were stained with BrdU antibody (1:200, Invitrogen) after being incubated with BrdU solution (1:100, Invitrogen) for 12 h, and then Alexa Fluor 568-conjugated secondary antibody were used to stain the cells for 1 h at room temperature. Finally, slides were mounted with Vectashield mounting medium containing DAPI (Vector Laboratories). The percentage of BrdU-positive cell number in total cell number was calculated. Three independent samples of each experimental group were used for BrdU assay and BrdU-positive and total cell numbers were counted in 10 images per subject.

### DSS-induced mouse colitis and treatment with PDLSCs

Female C57BL/6J (JAX #000664) mice were purchased from the Jackson Laboratory (Bar Harbor, ME, USA). Acute colitis was induced in 8-week-old C57BL/6J mice with 3% (w/v) DSS (MP Biochemicals) in the drinking water for 10 days^43^. A total of 2.0 × 10^6^ untreated PDLSCs or Tet1/Tet2 siRNA-treated PDLSCs were intraperitoneally injected into the mice 3 days after initiation of DSS treatment. DAI was scored on a scale of 0–4 by evaluating the weight loss, stool consistency/diarrhea, and presence of fecal bleeding^19,43^. At day 10, mice were sacrificed by CO_2_ euthanasia, their colons were fixed with 4% PFA, and paraffin-embedded sections were prepared for hematoxylin and eosin staining. Histological scores were blindly determined as previously described^19^. All animal experiments were performed under institutionally approved protocols for the use of animal research (University of Pennsylvania IACUC #805478).

### Chromatin immunoprecipitation-qPCR assays

PDLSCs were cross-linked and used for each immunoprecipitation (IP). ChIP was performed using the Millipore ChIP Kit according to the manufacturer’s protocol. Branson sonifier was used to shear the chromatin. For precipitating DNA–protein complexes, Tet1 and Tet2 antibodies were used and non-specific serum immunoglobulin G was used as an isotype control. Percentage input was determined by removing an aliquot of sheared chromatin prior to IP and comparing amplification of this DNA to amplification of the precipitated chromatin. MethPrimer software was used to predict the presence of CpG islands in the *DKK-1* promoter.

### Hydroxymethylated DNA immunoprecipitation

IP of 5-hmC was performed using an Active Motif hMeDIP Kit. A Branson sonicator was used to sonicate DNA into short fragments (100 to 1000 base pairs (bp)), after which it was heat denatured at 95 °C for 10 min. Sonicated DNA (1 μg) was immunoprecipitated with 2.5 μg of mouse anti-5-hmC antibody (Active Motif, 1 μg/μl) and was incubated overnight at 4 °C. Then, the magnetic beads were added to the DNA–antibody mixture and isolation of immunoprecipitated DNA was performed according to the kit instructions. SYBR^®^ Green Supermix (Bio-Rad) on a Bio-Rad CFX96 Real-Time system was used to perform qPCR, as indicated by the manufacturer’s protocol. The amount of DNA used in the IP reaction was calculated for the percentage of enrichment.

### Methylation-specific PCR and OxBS-sequencing

OxBS-sequencing was used to assess DKK-1 methylation status. A highly selective chemical oxidizes 5-hmC to 5-formylcytosine (5fC) and then bisulfite treatment deformylated and deaminated 5fC to uracil, appears as thymine (T) in sequencing analysis. 5-mC is not determinate and appears as a cytosine (C) in sequencing analysis. Oxidative bisulfite conversion, which is able to produce an accurate readout distinguishing 5-mC from 5-hmC, was performed using a TrueMethyl™ Kit (Cambridge Epigenetix, UK), according to the manufacturer’s instructions^44^. For methylation-specific PCR, pretreated DNA was amplified with methylation-specific primers for site 1 and site 2 of DKK-1 and were sequenced as previously reported by us^10^.

### Statistical analysis

All results are presented as the mean and standard deviation (mean ± SD) for *n* from 3 to 8. Independent unpaired two-tailed Student’s *t* tests were used for comparing between two groups. One-way analysis of variance was used for comparing between more than two groups. *P* values < 0.05 were considered statistically significant.

## Supplementary information

Supplementary material
