# Supplementary material for: Inhibition of Tet1- and Tet2-mediated DNA demethylation promotes immunomodulation of periodontal ligament stem cells
Source: Cell Death Dis. 2019 Oct 14;10(10):780. doi: 10.1038/s41419-019-2025-z (PMC6791886; doi:10.1038/s41419-019-2025-z)
Supplement: Supplementary file 1 — Supplementary material [file 41419_2019_2025_MOESM1_ESM.docx]

**Supplementary materials**


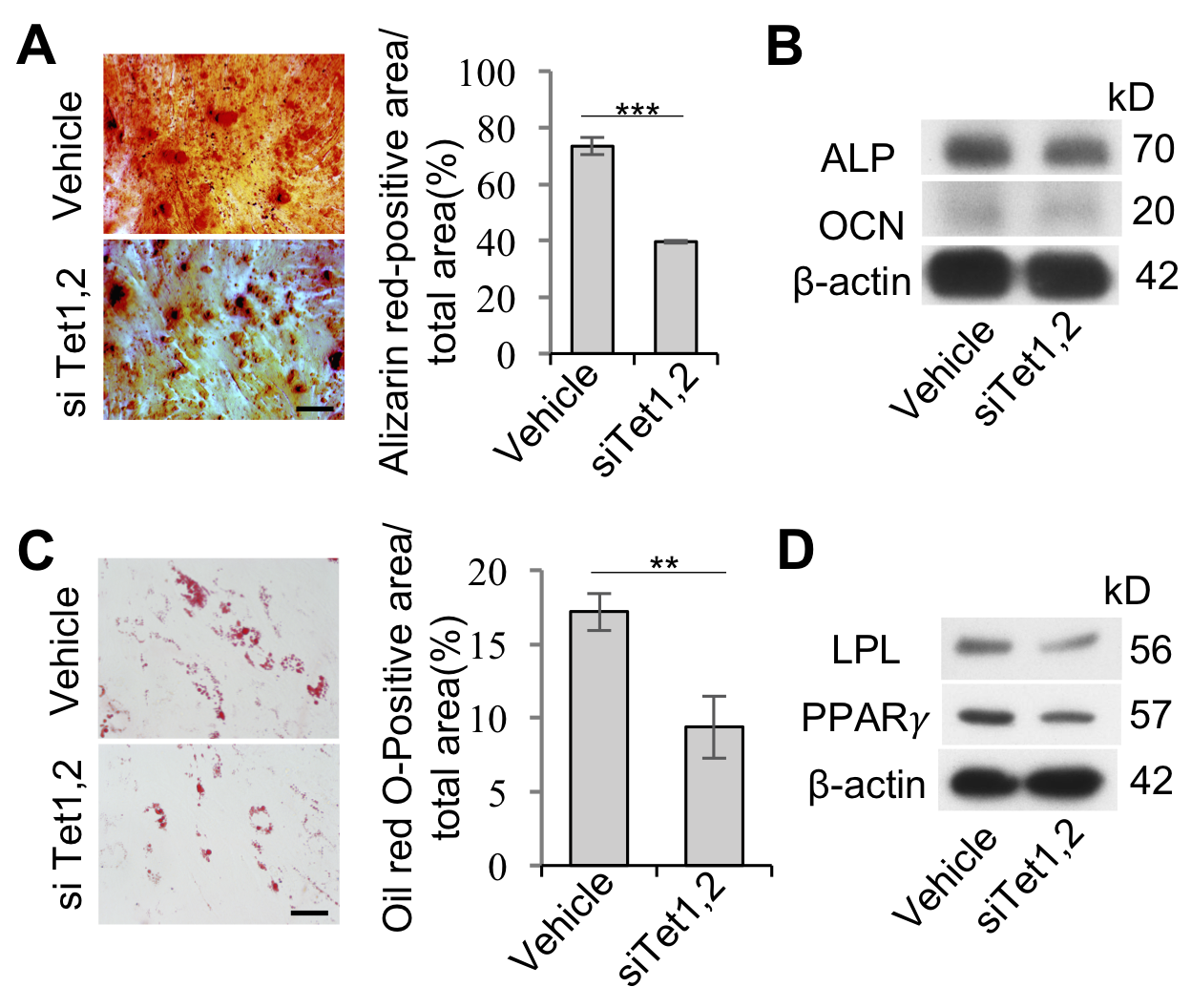


**Supplementary Fig. 1 Inhibition of Tet1 and Tet2 reduces PDLSC osteogenic and adipogenic differentiation.** (**a and b)** Tet1 and Tet2 siRNA knockdown reduced mineralized nodule formation in PDLSCs as assessed by alizarin red staining **(a)** and decreased expression of osteogenic markers *ALP* and *OCN* as assessed by Western blotting **(b)**. Scale bar in (a), 50 μm. (**c and d)** Tet1 and Tet2 siRNA knockdown reduced lipid droplet formation in PDLSCs as assessed by Oil-red O staining **(c)** and decreased expression of adipogenic markers *LPL* and *PPARγ* as assessed by Western blotting **(d)**. Scale bar in (c), 50 μm. ****p*<0.001, ***p*<0.01; *p* values calculated using two-tailed Student’s *t* test (mean ± S.D.).


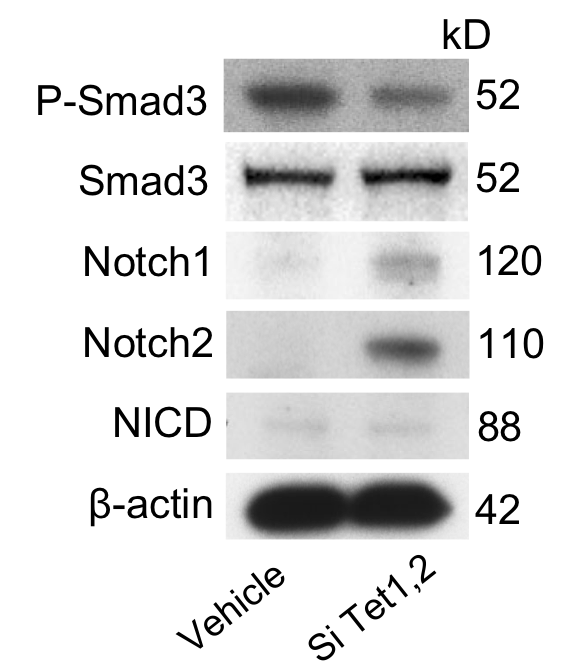


**Supplementary Fig. 2** Western blot analysis showed the expression levels of TGF-β and Notch signaling in Tet1 and Tet2 siRNA-treated hPDLSCs.
